# Temozolomide and Capecitabine Treatment for an Aggressive Somatotroph Pituitary Tumor: A Case Report and Literature Review

**DOI:** 10.3389/fonc.2022.916982

**Published:** 2022-05-26

**Authors:** Atsushi Ishida, Hiroki Shichi, Hidenori Fukuoka, Hideki Shiramizu, Naoko Inoshita, Shozo Yamada

**Affiliations:** ^1^ Hypothalamic and Pituitary Center, Moriyama Memorial Hospital, Tokyo, Japan; ^2^ Division of Diabetes and Endocrinology, Kobe University Graduate School of Medicine, Kobe, Japan; ^3^ Department of Pathology, Moriyama Memorial Hospital, Tokyo, Japan

**Keywords:** aggressive somatotroph pituitary tumor, temozolomide, capecitabine, O6-methylguanine DNA methyltransferase, 3D spheroid *ex vivo* assay

## Abstract

Aggressive somatotroph pituitary tumor that causes acromegaly is extremely rare and resists conventional treatments such as multiple surgeries, radiotherapies, and various types of somatostatin analogs. Here, we propose a novel treatment option for these rare cases by discussing our case and reviewing the literature. We experienced an aggressive somatotroph tumor in a 52-year-old woman with acromegaly. Not only could a complete remission of growth hormone (GH) and insulin-like growth factor-1 (IGF-1) not be obtained, but the tumor continued to grow and eventually recurred around the brainstem despite multidisciplinary treatments. We employed immunohistochemistry and a three-dimensional (3D) spheroid *ex vivo* assay to determine the best treatment option for this case. Although histology showed strong *O* ^6^-methylguanine DNA methyltransferase expression and high Ki-67 labeling index (22%), temozolomide (TMZ) combined with capecitabine (CAPTEM) treatment was performed based on the results of the patient-derived 3D spheroid *ex vivo* assay, which predicted more effective treatment with CAPTEM than with TMZ alone. Consequently, GH and IGF-1 levels were restored to normal range with remarkable tumor shrinkage after CAPTEM treatment. To the best of our knowledge, there have been even very few reports describing successful treatment for such aggressive and refractory somatotroph tumors and this is the first report showing the effectiveness of CAPTEM on refractory somatotroph tumor both *ex vivo* and *in vivo*.

## Introduction

Acromegaly is an endocrine disorder characterized by hypersecretion of growth hormone (GH) from pituitary tumors (1). Acromegaly displays significant comorbidity and an increased mortality if not successfully treated, such as hypertension, impaired glucose tolerance, cardiovascular diseases, and malignancy (2). Transsphenoidal surgery (TSS) is the first-line treatment because it has been shown to improve the chance of complete endocrinological remission (CR) (3, 4). Even for those without CR after TSS, acromegaly usually is well controlled with various types of medical therapies including somatostatin receptor ligands, dopamine antagonists, and GH receptor antagonists (5, 6). Therefore, compared with other functioning pituitary tumors, it is extremely rare for somatotroph pituitary tumors causing acromegaly to become aggressive (7, 8). Thus, there have been very few reports concerning refractory somatotroph tumors. Temozolomide (TMZ) has been considered the first choice of treatment for aggressive or malignant pituitary tumors (9). Many papers have shown that the degree of expression of *O* ^6^-methylguanine DNA methyltransferase (MGMT) is inversely proportional to the effect of TMZ (10, 11). TMZ in combination with capecitabine, a prodrug of 5-fluorouracil (5-FU), (CAPTEM) has recently been developed as one of treatment options for MGMT-positive and TMZ refractory aggressive pituitary tumors (12). Here, we report an aggressive somatotroph pituitary tumor in which CAPTEM was very effective both *ex vivo* and *in vivo* and include a brief review of the literature.

## Case Description

**Figure 1:**
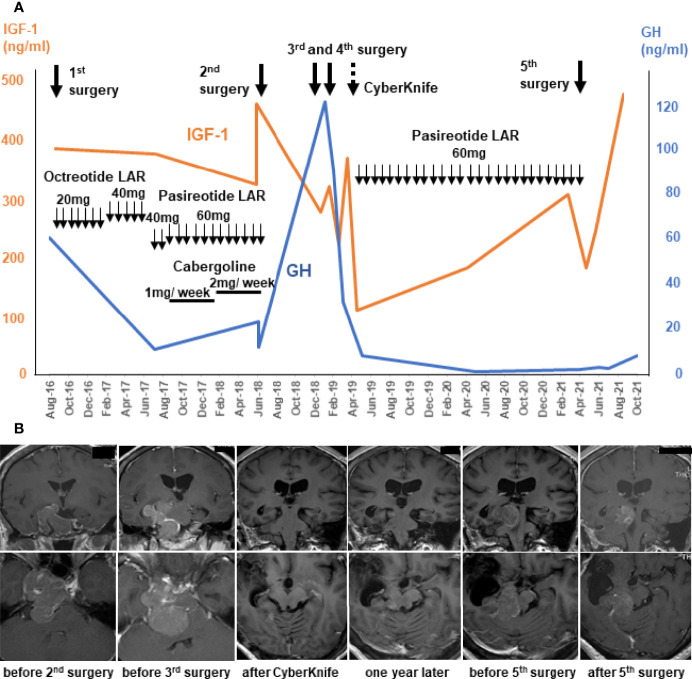
Clinical course and management of the patient using multimodal treatment strategy until temozolomide and capecitabine (CAPTEM). Serum levels of growth hormone (GH; blue lines) and insulin-like growth factor-1 (IGF-1; orange lines) before CAPTEM **(A)**. Gadolinium-enhanced MR T1 images show that the tumor initially extending laterally to the right and posteriorly, and finally localized right-dorsal part of the brainstem. The upper row is coronal images and the lower row is axial images **(B)**.

A 47-year-old woman presented with right-side blurred vision and right quadrant hemianopsia,and was diagnosed with acromegaly based on endocrine examinations. Magnetic resonance imaging (MRI) revealed a large pituitary tumor in the sella turcica extending to suprasellar region and right cavernous sinus (CS). TSS was performed at another hospital, and resulted in partial removal. She was initially treated with octreotide long-acting release (LAR), but it was ineffective even with increasing doses (**Figure 1A**) and was replaced with increasing doses of pasireotide LAR along with cabergoline (dose was up to 2 mg weekly) (**Figure 1A**. Neither endocrinological improvement nor tumor shrinkage was obtained despite the use of these various medications for two years. She was then referred to our hospital to seek another opinion in June 2018. Her serum GH level was 23.1 ng/ml and IGF-1 level was 324 ng/ml (age and sex adjusted IGF-1 reference range: 83-221 ng/ml) without any other anterior pituitary hormone deficiency. MRI revealed a large sellar mass invading the right CS with complete internal carotid artery (ICA) encasement, which was equivalent to Knosp grade 4; in addition, the tumor extended posteriorly, compressing the brainstem (**Figure 1B**).

The repeat surgery performed at our hospital resulted in partial tumor removal (approximatey 70% tumor reduction), because the tumor was in proximity to vital structres such as ICA, cranial nerves, and the brainstem. Histology showed a sparsely granulated somatotroph tumor with 3.2% Ki-67 labeling index (LI). Both SSTR2A and SSTR5 staining by immunohistochemistry were 3+ suggesting effectiveness of any somatostatin analogs. However, even with the subsequent medical therapy, the tumor continued growing and serum GH levels continued to rise (**Figure 1**).

Additional surgery was scheduled after 6 months, because she was at risk of imminent tentorial herniation caused by the enlarged tumor and the compression of the brainstem was even more severe than before. Emergent surgery (simultaneous right frontotemporal craniotomy and TSS) was performed, but the tumor near the brainstem was not able to be removed. Three weeks later, right petrosal approach was performed for the left tumor. A significant amount of tumor remained and CyberKnife treatment was administered. These intensive treatments worked and tumor volume and serum levels of GH and IGF-1 drastically decreased (**Figure 1A**). The pathological study of the specimen revealed that GH-positive cells were significantly reduced compared with the previous surgery, and Ki-67 LI was elevated up to 15.1%, suggesting that the tumor had become more undifferentiated and aggressive. Thereafter, pasireotide treatment controlled her disease well for a period.

**Figure 2:**
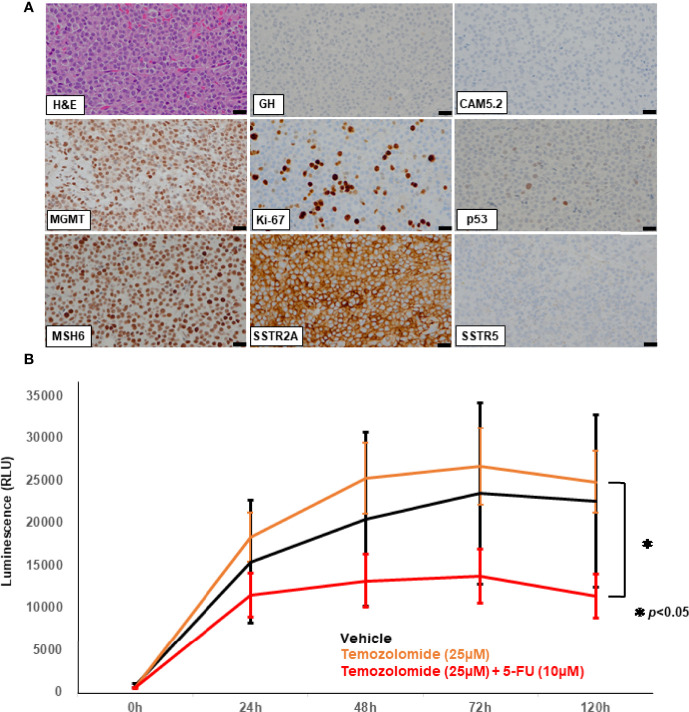
Hematoxylin and eosin (HE) staining and immunohistochemical studies of the specimen from the 5^th^ surgery **(A)** using the following antibodies: growth hormone (GH), CAM5.2, *O* ^6^-methylguanine-DNA-methyltransferase (MGMT), Ki-67, p53, MutS Homolog 6 (MSH6), somatostatin receptor (SSTR) 2A, SSTR5. Scale bar represents 20 mm and applies to all the pictures **(A)**. Patient-derived *ex vivo* three-dimensional (3D) spheroid culture assay **(B)**. Cell Viability Assay is shown in the bar graph. X-axis represents the time after drug treatment (hours) while the y-axis represents the value of luminescence (RLU). The luminescence readout is directly proportional to the number of viable cells in culture. Temozolomide (TMZ) combined with 5-fluorouracil (5-FU) (red line) had significantly more effect than TMZ alone (orange line). TMZ alone (orange line) showed no effect compared to the vehicle treatment (black line). 50% inhibition concentration (IC50) was used as a reference for setting drug concentration conditions. The *p*-value of the two-sided test less than 0.05 was considered to represent statistical significance. RLU: relative light unit. *p < 0.05.

However, the tumor reappeared in the dorsal part of the right midbrain and serum IGF-1 levels increased one year after (**Figure 1A**). Serial MRI in this period revealed that the tumor volume became 30 times larger in 5 months (**Figure 1B**). A very recent report suggested that a tumor growth rate ≥2.2% per month could be a criterion for aggressive pituitary tumors (13). Considering these criteria, this tumor was regarded as rather aggresive. The fifth surgery was performed for the tumor, but only small part was removed because it was deep and adhered to the brainstem (**Figure 1B**). Pathological examination of the resected fragments confirmed the pleomorphic nature of the majority of the tumor cells; only scattered cells were immunopositive for GH antibody (<1%) (**Figure 2A**); CAM5.2 was almost negative, and Ki-67 LI was further increased up to 22%. In addition, MGMT was strongly positive, and MutS homolog 6 (MSH6) was positive, in almost all tumor cells. P53 was sporadic but strongly positive. Although SSTR5 was previously 3+, it was almost negative this time, while SSTR2 remained positive (3+) (**Figure 2A**).

**Figure 3:**
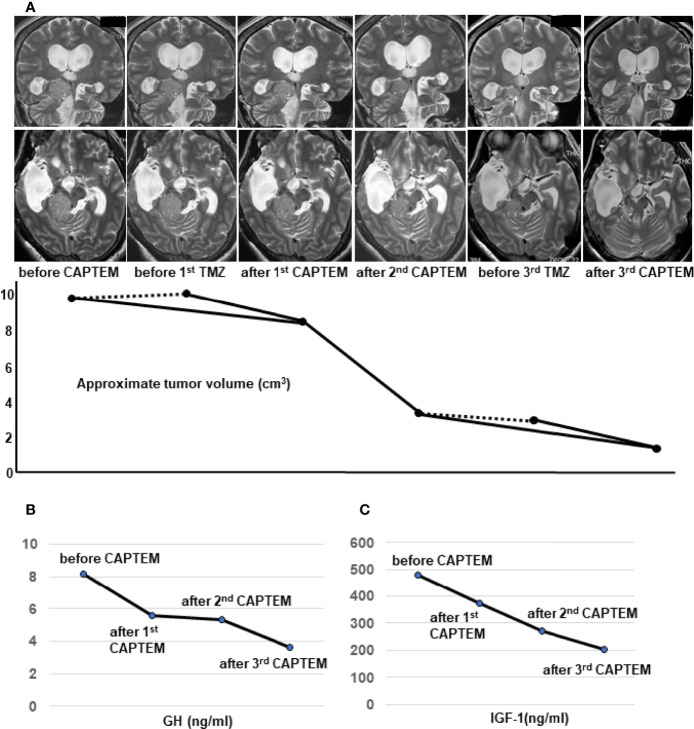
Temozolomide and capecitabine (CAPTEM) had a significant effect on reduction of the tumor volume and serum levels of growth hormone (GH) and insulin-like growth factor (IGF-1). Thin-slices of plain T2 images were used to show the dramatic changes of the tumor volume by CAPTEM treatment **(A)**. The upper row shows coronal images and the lower row shows axial images **(A)**. Two orthogonal diameters of the tumor were measured in the coronal and axial T2-weighted images. The average of the 4 diameters was assumed as the diameter of an approximated sphere. Then, the formula of a sphere volume; 4/3πr^3^ was used to calculate the approximate tumor volume **(A)**. MRI just before TMZ administration did not show the volume reduction (dotted lines) but MRI just after TMZ showed considerable shrinkage (solid lines) **(A)**. CAPTEM had a significant effect on reduction of morning serum GH **(B)** and IGF-1 **(C)** levels. IGF-1 normal range for the patient’s age and sex is 78-213 ng/ml.

To evaluate the direct effect of TMZ on this tumor, we employed an *ex vivo* three-dimensional (3D) spheroid culture assay of the resected tumor, which is thought to mimic more realistic environment for the tumors than two-dimensional (2D) cultures, as described previously (14, 15). In this assay, we measured cell viability using RealTime-Glo MT Cell Viability Assay (Promega). The results showed that TMZ in combination with 5-FU reduced cell viability by 50% compared to vehicle treatment (*p*<0.05), while no reduction was seen with TMZ alone, indicating that CAPTEM treatment would be more effective than TMZ monotherapy for the present case (**Figure 2B**). GH concentration of the cultured medium was also assessed comparing vehicle, TMZ alone, and TMZ +5-FU treatment. The concentration of GH in the medium was too low to compare among those groups (data not shown), which reflects the low level of serum GH when the tumor was removed (**Figure 1A**). Following this result, CAPTEM (capecitabine 750 mg/m^2^ twice daily on days 1–14 and TMZ 200 mg/m^2^ once daily on days 10–14) treatment was administered in combination for 2 weeks, followed by 2 weeks off, as previously described (14). CAPTEM treatment showed a significant reduction in tumor size, and it reduced to one seventh of the initial size after the 3^rd^ round of CAPTEM. MR T2 images obtained after capecitabine administration (prior to TMZ administration) (day 10) showed no tumor shrinkage, but remarkable reduction of the tumor volume was visible on MRI obtained just after TMZ administration (day 15) (**Figure 3A**), suggesting that the tumor shrinkage was not caused by capecitabine itself, but by the use of TMZ after capecitabine. Serum IGF-1 and GH levels were also decreased by CAPTEM treatment (**Figures 3B, C**). CAPTEM was highly tolerable for the patient with no noteworthy side effects. Before CAPTEM, the patient suffered from intractable seizures even with intensive anti-epileptic drugs treatment, and her conscious level was too low to even consume food orally. Now, her conscious level is almost clear, with no epileptic attack. She is currently undergoing rehabilitation to ensure return to normal life.

## Discussion

Due to the development of endoscopic TSS, a high percentage of acromegaly patients can gain CR by surgery alone (3, 4). However, tumors extending laterally, especially with Knosp grade 4 like this case, cannot be completely removed. Even following radiosurgery and medical treatments is not always effective because of exacerbating aggressive nature. In this case, Ki-67 LI increased (from 3% to 22%) with each recurrence, while GH-positive cells decreased (from 80% to almost zero). Serum GH level also declined dramatically (**Figure 1**). These changed in tumor characteristics suggest that the tumor may have gradually dedifferentiated and became more aggressive. Aggressive somatotroph pituitary tumors are extremely rare and its mechanism of malignant transformation remains unclear. Vekaria et al. recently reviewed 25 reports of pituitary carcinoma in acromegaly (16). They concluded that genetic mutations driving somatotroph carcinoma tumorigenesis and effective targeted therapies were largely unknown (16).

The clinical experience with TMZ in aggressive pituitary tumors has grown since the initial successful reports were published in 2006 (17). A recent trial examining the effect of MGMT expression in aggressive pituitary tumors showed that TMZ responders and non-responders had a median MGMT staining of 9% and 93%, respectively (10); therefore, TMZ remains a viable option in aggressive somatotroph pituitary tumors and appears to be more effective in tumors that lack expression of MGMT. In contrast with our case however, the majority of these cases were corticotroph tumors or lactotroph tumors (9, 18) and reports of TMZ use in treating refractory somatotroph tumors are very limited (6, 16). We meticulously reviewed all published reports and our own experiences of no less than 1500 somatotroph tumors. There have been 11 cases, including our two, describing both TMZ treatment outcomes and the degree of MGMT expression, in aggressive or malignant somatotroph tumors; the summary of these cases is shown in **Table 1** (9, 10, 19–22). Several previous studies showed the correlation between MGMT promoter methylation and protein expressions in IHC (23, 24), and it is thought that unmethylated MGMT promotor most leads to strong expressions in IHC (23, 24). Two cases which analyzed the promoter methylation status reported unmethylated MGMT promoters (20, 22); and nine cases reported strong expression of MGMT. This is in direct contrast to aggressive corticotroph tumors which are usually weakly positive or negative in MGMT IHC according to our own experiences and the available literature (25, 26). The reasons why aggressive or malignant somatotroph tumors tend to have strong MGMT expression remains unclear. It is possible that MGMT (DNA repair enzyme) is one of the functions of normal cells suppressing or preventing the canceration of cells, and its preservation (MGMT positivity) may indicate that the degree of dedifferentiation is less than that of disappearing tumors. Moreover, it may be one of the reasons why is it extremely unusual for somatotroph tumors to develop into aggressive or cancerous tumors compared to other functional tumors like corticotroph or lactotroph tumors. Of the cases with strong MGMT expression, TMZ monotherapy was only effective in 3 out of 8 cases (except the present case). In contrast, the two cases with weak expressions of MGMT responded well to TMZ, and the present case showed a remarkable response to CAPTEM treatment (**Table 1**).

**Table 1:** O6-methylguanine DNA methyltransferase (MGMT) status and temozolomide treatment in aggressive or malignant somatotroph tumors.

Study	Sex/age	Extent of aggressiveness	Postoperative treatments	MGMT status	Temozolomide dose (mg/m^2^)	outcome
Hagen et al (18)	F/48	LN metastasis	RT, octreotide, CAB	50% in IHC	Conventional, total 23 cycle	Complete regression
McCormack et al. (9)	M/48	Extremely invasive	RT, octreotide, CAB	Positive in IHC	150x 5/28 for 3 cycles	No improvement
Ceccato et al. (19)	F/39	Locally aggressive	CAB	unmethylated promoter	150-200x 5/28 for 3 cycles	45% increase in tumor volume
Bengtsson et al. (10)	F/31	Locally aggressive	RT, SSA	9-100% in IHC	150-200x 5/28 for 6 cycles	50% tumor regression
Bengtsson et al. (10)	M/33	Locally aggressive	DA, SSA	10% in IHC	150-200x 5/28 for 3 cycles	35% tumor regression
Bengtsson et al. (10)	M/46	Liver metastasis	RT, SSA, PEG	90% in IHC	Conventional, total 2 cycle	Progressive growth
Bengtsson et al. (10)	F/40	Cerebral metastasis	RT, DA	9% in IHC	150-200x 5/28 for 6 cycles	Complete regression
Batisse et al. (20)	M/47	Locally aggressive	SSA	90% in IHC	150-200x 5/28 for 5 cycles	No improvement
Chandler et al. (21)	F/59	Vertebral metastasis	RT	unmethylated promoter	Details are not available	Radiological improvement of metastatic lesion
Our previous experience	M/66	Locally aggressive	RT, octreotide	90% in IHC	150-200x 5/28 for 3 cycles	Increase in tumor volume
Present case	F/52	Locally aggressive	RT, octreotide, pasireotide	90% in IHC	with capecitabine for 3 cycles	85% tumor regression.

LN, lymph node; RT, radiotherapy; CAB, cabergoline; SSA, somatostatin analogue; PEG, pegvisomant; DA, dopamine agonist; IHC, immunohistochemistry.

A recent meta-analysis described the safety and efficacy of CAPTEM in the treatment of advanced neuroendocrine tumors (27). In addition, CAPTEM treatment for aggressive pituitary tumors has been reported in several cases (25, 26). Capecitabine is an antimetabolite that incorporates 5-fluorodeoxyuridine triphosphate into DNA, leading to the attenuation of MGMT repair activity through thymidylate synthase inhibition and reduction in thymidine level, thereby enhancing the alkylating effect of TMZ (28). TMZ is the only realistic option for aggressive pituitary tumors, but a positive effect of TMZ has been observed in only 47% of cases (29). MGMT expression levels are the most reliable predictors of TMZ response (9, 10), although the relationship between the presence or absence of MGMT expression and the effect of TMZ is still controversial. Therefore, it was expected that TMZ might not work in this case where MGMT was strongly positive.

Patient-derived 3D culture is a promising drug-screening tool that has been used for various refractory neoplasms (30). These culture models have an environment that more closely mimics various solid tumors more than 2D culture models (31). In this case we compared TMZ alone and in combination with 5-FU using 3D culture. Consequently, the CAPTEM-mimicked assessment revealed a significantly greater effect on reducing cell viability than TMZ monotherapy. The result was extremely helpful for us to initiate CAPTEM regimen for the patient from the beginning. To the best of our knowledge, this is the first report to prove CAPTEM treatment effect in real world in refractory aggressive somatotroph tumor with strong MGMT expression.

In our view, CAPTEM treatment, not TMZ monotherapy, may be the first choice for aggressive somatotroph tumors which typically show strong MGMT expression. In addition, the *ex vivo* 3D culture assay system may be a useful tool to help clinicians to choose between TMZ monotherapy or CAPTEM treatment with more confidence.

## Data Availability Statement

The raw data supporting the conclusions of this article will be made available by the authors, without undue reservation.

## Ethics Statement

The studies involving human participants were reviewed and approved by Moriyama Memorial Hospital. The patients/participants provided their written informed consent to participate in this study.

## Author Contributions

AI and SY had the idea. Material preparation, data collection and analysis were performed by AI, HS, HF, HS, NI, and SY. The first draft of the manuscript was written by AI. SY and HF supervised the manuscript drafting. All authors read and approved the final manuscript.

## Conflict of Interest

The authors declare that the research was conducted in the absence of any commercial or financial relationships that could be construed as a potential conflict of interest.

## Publisher’s Note

All claims expressed in this article are solely those of the authors and do not necessarily represent those of their affiliated organizations, or those of the publisher, the editors and the reviewers. Any product that may be evaluated in this article, or claim that may be made by its manufacturer, is not guaranteed or endorsed by the publisher.
